# Sanguinarine Induces Apoptosis in Papillary Thyroid Cancer Cells via Generation of Reactive Oxygen Species

**DOI:** 10.3390/molecules25051229

**Published:** 2020-03-09

**Authors:** Abdul Q. Khan, Elham A. N. Mohamed, Ishrat Hakeem, Aneeza Nazeer, Shilpa Kuttikrishnan, Kirti S. Prabhu, Kodappully S. Siveen, Zafar Nawaz, Aamir Ahmad, Hatem Zayed, Shahab Uddin

**Affiliations:** 1 Translational Research Institute, Academic Health System, Hamad Medical Corporation, Doha 3050, Qatar; AKhan42@hamad.qa (A.Q.K.); EAhmed16@hamad.qa (E.A.N.M.); ishrathakeem@yahoo.com (I.H.); Aneezanazeer@gmail.com (A.N.); SKuttikrishnan@hamad.qa (S.K.); KPrabhu@hamad.qa (K.S.P.); SSivaraman@hamad.qa (K.S.S.); 2 Department of Lab Medicine and Pathology, Hamad Medical Corporation, Doha 3050, Qatar; Znawaz@hamad.qa; 3 Department of Biomedical Sciences, College of Health Sciences, QU Health, Qatar University, Doha 3050, Qatar; hatem.zayed@qu.edu.qa; 4 Department of Medicine, University of Alabama at Birmingham, Birmingham, AL 35205, USA

**Keywords:** thyroid cancer, cell proliferation, sanguinarine, cisplatin, STAT3, apoptosis, autophagy

## Abstract

Sanguinarine (SNG), a natural compound with an array of pharmacological activities, has promising therapeutic potential against a number of pathological conditions, including malignancies. In the present study, we have investigated the antiproliferative potential of SNG against two well-characterized papillary thyroid cancer (PTC) cell lines, BCPAP and TPC-1. SNG significantly inhibited cell proliferation of PTC cells in a dose and time-dependent manner. Western blot analysis revealed that SNG markedly attenuated deregulated expression of p-STAT3, without affecting total STAT3, and inhibited growth of PTC via activation of apoptotic and autophagy signaling cascade, as SNG treatment of PTC cells led to the activation of caspase-3 and caspase-8; cleavage of PARP and activation of autophagy markers. Further, SNG-mediated anticancer effects in PTC cells involved the generation of reactive oxygen species (ROS) as N-acetyl cysteine (NAC), an inhibitor of ROS, prevented SNG-mediated antiproliferative, apoptosis and autophagy inducing action. Interestingly, SNG also sensitized PTC cells to chemotherapeutic drug cisplatin, which was inhibited by NAC. Finally, SNG suppressed the growth of PTC thyrospheres and downregulated stemness markers ALDH2 and SOX2. Altogether, the findings of the current study suggest that SNG has anticancer potential against PTC cells as well its derived cancer stem-like cells, most likely via inactivation of STAT3 and its associated signaling molecules.

## 1. Introduction

Cancer is a deadly disease with many challenges and remains a leading cause of death worldwide. Future predictions about this disease are even more frightening in terms of morbidity and mortality [1,2]. It is a complex disease marked by genetic/epigenetic deregulations and aberrant signaling mechanisms. A number of preclinical and clinical reports have shown that deregulated signaling pathways are the primary cancer characteristics [3,4,5,6]. It is well established that signaling pathways are integral for cell survival, growth and maintenance of normal homeostasis and other important biological activities. Once deregulated, they lead to the onset of a number of molecular, cellular and biological changes manifested by various human diseases, including cancer [4,7].

Aberrant or abnormal expression and functioning of various survival pathways, including Janus kinase/signal transducer and activator of transcription (JAK/STAT) and phosphoinositide 3-kinase (P13K/AKT) pathways, has been implicated in carcinogenesis and associated challenges, such as stemness, resistance and recurrence. It has now become evident that dysregulated STAT3, vital for the expression and functioning of genes required for cell survival and development, including immunity and stemness, is a crucial target to overcome the cancer pathogenesis and related challenges [8,9,10,11]. The role of STAT3 in thyroid malignancies is relatively less studied, however, there are data from some in vitro as well as in vivo studies [12,13,14,15], including the recent investigations on how STAT3 plays a critical role in noncoding RNA-mediated alterations related to the thyroid cancer pathogenesis [16,17,18]. In addition, the critical role of STAT3 in the development of both solid and hematological human malignancies has been reported by many research groups [19,20,21]. STAT3 is, therefore, a legitimate target for cancer management and therapy [20,22]. 

**Figure 1:**
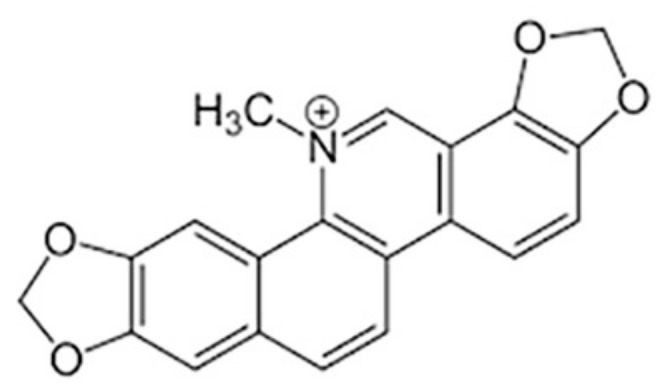
Chemical structure of sanguinarine.

Considering the above facts, we have designed the current study to investigate whether sanguinarine (SNG)-induced anticancer and antiproliferative effects on papillary thyroid cancer (PTC) involve the downregulation of STAT3 and associated signaling molecules. PTC is the most prevalent thyroid cancer with increasing incidence, and generally good prognosis but increased recurrence [23]. In the current study, we evaluated the effect of a natural compound, SNG (Figure 1), as an anticancer agent against PTC cells. SNG is generally obtained from the roots of *Sanguinaria canadensis* and chemically categorized as benzophenanthridine alkaloid [19,24]. In plants, biosynthesis of SNG involves the combination of 4-hydroxyphenyl-acetaldehyde and dopamine to form norcoclaurine; this is followed by the addition of methyl hydroxyl groups. The final step in the synthesis of SNG involves the conversion of precursor dihydrosanguinarine to SNG by the enzyme dihydrobenzophenanthridine oxidase. 

A number of studies have shown the therapeutic potential of SNG over a range of human pathological and toxicological conditions including cancer; for example, lung cancer [25], cervical cancer [26], gastric cancer [27,28], liver cancer [29,30], multiple myeloma [19], acute lymphoblastic leukemia [31], prostate cancer [32], colorectal cancer [33], ovary cancer [34] and pancreatic cancer [35]. Recently Achkar et al. [36] extensively reviewed the anticancer features of SNG and, additionally, antioxidant/anti-inflammatory [37,38,39], antifungal [40,41], antibacterial [42,43], anthelmintic [44] and other pharmacological activities of SNG have also been reported. SNG has been shown to suppress stemness of pancreatic cancer stem cells [45] and, interestingly, also to exert anticarcinogenic potential via modulating expression and functioning of noncoding RNAs [28,34]. Reactive oxygen species (ROS) production, among others, has been considered as a prime underlying mechanism for SNG [30,46,47]. Furthermore, SNG has been found to sensitize cancer cells to anticancer drugs through attenuated resistance and stemness [35,48,49].

To the best of our knowledge, this is the first investigation showing the anticancer potential of SNG in thyroid cancer. In the current study, we investigated the anticancer potential of SNG against PTC cell lines BCPAP and TPC-1 and found that SNG had strong anticancer potential against PTC, as it inhibits cell proliferation and growth. SNG also inhibited the cancer stemness potential of PTC cells and, additionally, sensitized PTC cells to anticancer drug cisplatin. Our data also showed that ROS had an important role in SNG-mediated death of the PTC cells.

## 2. Results

### 2.1. SNG Suppresses Proliferation of PTC Cells

**Figure 2:**
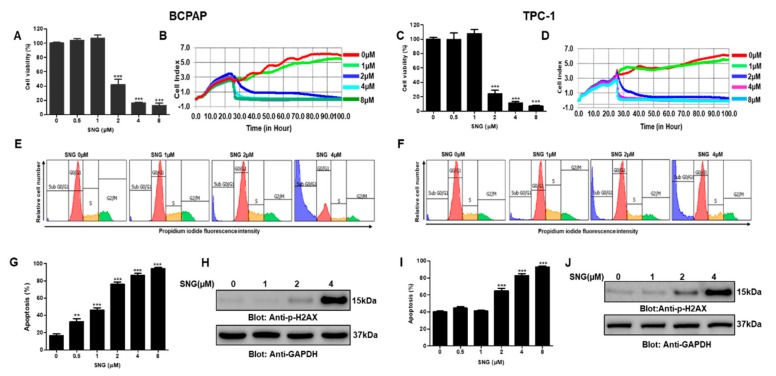
Sanguinarine (SNG)-mediated cytotoxic effects in papillary thyroid cancer (PTC) cells. BCPAP (**A**) and TPC-1 (**C**) cells were treated with 0 µM, 0.5 µM, 1 µM, 2µM, 4 µM and 8 µM SNG for 24 h. Cell proliferation assay was performed using the CCK-8 kit, as described in materials and methods. Values are expressed as the mean +/− SD (standard deviation) of at least six replicates (*n* = 6) with *p* value *** *p* < 0.001. Real-time cell proliferation (cell index) of PTC cells in response to SNG: BCPAP (**B**) and TPC-1 (**D**) cells were seeded on electrodes containing E-Plate VIEW 16 (ACEA Biosciences, Inc.) and after 24 h, treated with different doses of SNG, as indicated, followed by monitoring of cell growth in real-time as described in materials and methods. SNG induced cell cycle arrest in PTC cells: BCPAP (**E**) and TPC-1 (**F**) cells were treated with indicated doses of SNG for 24 h and analyzed by flow cytometry to detect cell cycle fractions, as described in materials and methods. SNG-mediated induction of apoptosis in PTC cells: BCPAP (**G**) and TPC-1 (**I**) cells were treated with SNG, as indicated, for 24 h, subjected to annexin V and dead cell analysis according to the manufacturer’s instructions and then analyzed by Muse^®^ cell analyzer, as described in materials and methods. Values are represented as mean ± SD (standard deviation) with *p* < 0.001 (*n* = 3). SNG-mediated phosphorylation H2AX in PTC cell lines: BCPAP (**H**) and TPC-1 (**J**) cells were treated with SNG, as indicated, for 4 h, and cells were subsequently lysed and immunoblotted with anti-p H2AX antibody and then stripped and probed for GAPDH.

A series of experiments were performed to investigate the effect of varying doses of SNG on the growth and proliferation of PTC cells. BCPAP and TPC-1 cells were treated with gradient doses (0 μM, 0.5μM, 1 μM, 2 μM, 4 μM and 8 μM) of SNG for 24 h in 96 well plates and subjected to CCK-8 based cell proliferation assay kit, as described in materials and methods. Our data analysis showed that SNG effectively suppressed the proliferation of PTC cells BCPAP and TPC-1, as shown in Figure 2A,C, respectively. Further, we observed the IC50 of SNG to be in the range of 1–2 μM. Next, we wanted to assess the inhibitory potential of SNG on PTC cell proliferation in real-time and, therefore, used xCELLigence real-time cell analysis (RTCA). BCPAP and TPC1 cells were again treated with increasing doses of SNG, and data were acquired as described in materials and methods. SNG suppressed the proliferation index of BCPAP and TPC-1 cells in a dose-dependent manner as depicted in Figure 2B,D, respectively. Further, RTCA data also revealed that SNG treatment suppressed BCPAP cell migration, as shown in Supplementary Materials Figure S1A. We then investigated the effect of SNG treatment on cell cycle in PTC cells, and our data showed a markedly increased SubG0/G1 fraction of cell cycle in BCPAP and TPC-1 cells, as represented in Figure 2E,F and Supplementary Materials Figure S1B,C, respectively. We next investigated the apoptotic potential of SNG by using annexinV and dead cell kit by Muse^®^ cell analyzer and found significantly increased apoptosis in SNG-treated PTC cells, as shown in Figure 2G,I and Supplementary Materials Figure S1D,E.

**Figure 3:**
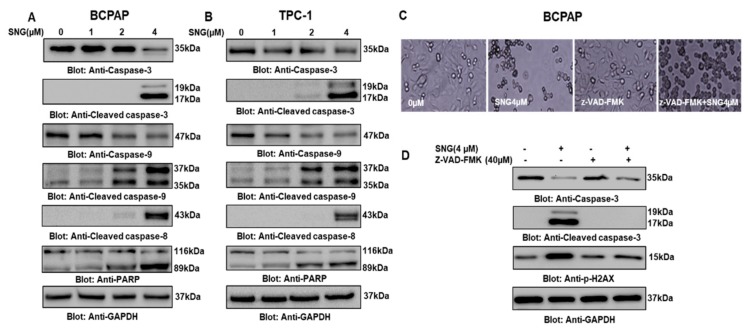
SNG-mediated activation of caspase cascade in PTC cells. SNG-induced activation of caspase-9, caspase-3, and cleavage of PARP in PTC cells. BCPAP (**A**) and TPC-1 (**B**) cells were treated with 1.0, 2.0 and 4 µM of SNG for 4 h. Cells were lysed, and proteins were separated on SDS-PAGE, transferred to a polyvinylidene difluoride (PVDF) membrane, and immunoblotted with antibodies against caspase-9, caspase-3, cleaved caspase-3, cleaved caspase-8, PARP and GAPDH. z-VAD-FMK, a pan-caspase inhibitor reversed SNG-mediated caspase activation: BCPAP cells (**C**) and (**D**) were pretreated with 40 μM z-VAD-FMK for 1 h and subsequently treated with 4 µM SNG for 4 h. Morphological changes were observed by microscopic analysis using the EVOS FLc cell imaging system from Invitrogen (Thermo Fisher Scientific). Cells were then lysed, and protein lysates resolved by SDS-PAGE, transferred to a PVDF membrane, and immunoblotted with antibodies against caspase-3, cleaved caspase-3 p-H2AX and GAPDH.

DNA damage has been shown to be a measure of apoptosis [50], therefore, we sought to analyze p-H2AX level, an important marker of DNA damage in PTC cells treated with 0 μM, 1 μM, 2 μM and 4 μM of SNG by Western blot. As shown in Figure 2H,J, increased expression of H2AX phosphorylation in response to SNG in BCPAP and TPC-1 cells was observed, supported by the densitometry data, as presented in Supplementary Materials Figure S1F,G, respectively. These data suggest that SNG-mediated apoptosis most likely may be due to double-strand DNA damage. We wanted to explore further the involvement of caspases in the SNG induced apoptosis in PTC cells. BCPAP and TPC-1 cells were treated with 0 μM, 1 μM, 2 μM and 4 μM of SNG for 4 h, and activation of caspases was analyzed by Western blot. A dose-dependent increase in the expression of cleaved caspases as well as cleaved PARP was observed when BCPAP and TPC-1 cells were subjected to SNG treatment (Figure 3A,B). These findings support our hypothesis that SNG-mediated growth inhibition of PTC cells occurs due to caspase-mediated apoptosis.

To further support the role of caspase-mediated apoptosis in response to SNG in PTC cells, BCPAP and TPC-1 cells were first pretreated with Z-VAD-FMK, a pan-caspase inhibitor, and subsequently treated with SNG followed by immunoblotting for caspase-3, cleaved caspase-3 and p-H2AX. Our data analysis revealed that Z-VAD-FMK treatment reversed SNG induced activation of caspases and phosphorylation of H2AX, which indicates SNG-mediated apoptosis involves activation of caspases in PTC cells (Figure 3C,D). We also performed CCK-8 based cell proliferation assay in BCPAP cells treated with caspase-9 inhibitor Z-LEHD-FMK in combination with SNG for 4 h, and observed a significant increase in cell viability of Z-LEHD-FMK + SNG treated group, as compared to only SNG treated cells (Supplementary Materials Figure S2A). In addition, treatment of BCPAP cells with inhibitors of caspase-9 (Z-LEHD-FMK) and caspase-8 (Z-IETD-FMK) also showed reversal of SNG-induced morphological changes and caspase-3 and cleaved caspase-3 activation (Supplementary Materials Figure S2B–E, respectively). Overall, these findings support the important role of caspase-mediated apoptosis in SNG induced growth inhibition of PTC cells.

### 2.2. Involvement of Reactive Oxygen Species (ROS) in SNG-Mediated Apoptosis of PTC Cells

**Figure 4:**
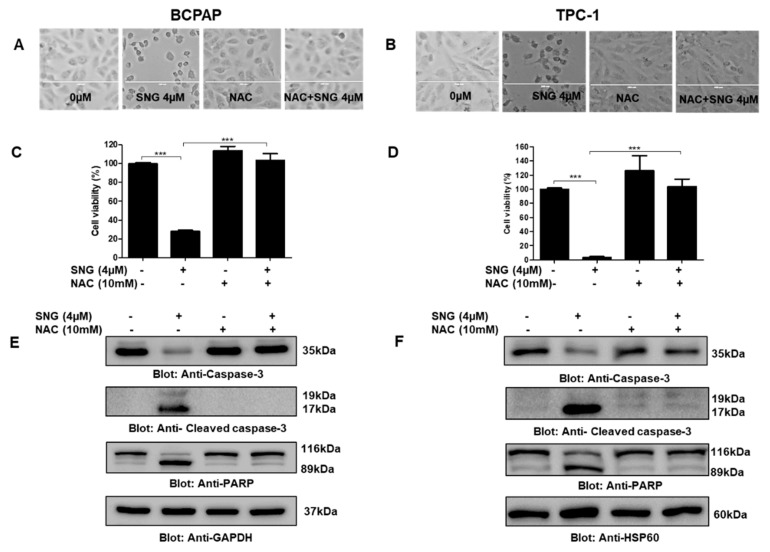
SNG-mediated cytotoxic action involves the generation of ROS in PTC cells. NAC pretreatment of PTC cells prevents SNG-induced morphological changes. BCPAP (**A**) and TPC-1 (**B**) cells were pretreated with 10 mM NAC for one hour and then treated with 4 µM SNG as indicated for 4 h, and morphological changes observed using the EVOS FLc cell imaging system (Thermo Fisher Scientific). NAC treatment reversed SNG-induced inhibition of cell viability. BCPAP (**C**) and TPC-1 (**D**) cells were pretreated with 10 mM NAC followed by subsequent treatment with 4 µM SNG for 24 h. CCK-8 was used to determine cell viability, as described in materials and methods. Values are expressed as mean +/− SD (standard deviation) of at least six replicates with *p* value *** *p* < 0.001 (*n* = 6). Effect of NAC treatment on SNG-induced markers of apoptosis: BCPAP (**E**) and TPC-1 (**F**) cells were pretreated with 10 mM NAC, followed by treatment with 4 µM SNG for 4 h. Cells were lysed, and proteins were separated on SDS-PAGE, transferred to a PVDF membrane, and immunoblotted with antibodies against caspase-3, cleaved caspase-3, PARP and GAPDH.

Since the role of ROS in various cancer therapeutic measures, including chemotherapy, is well known, we aimed to investigate the role of ROS in SNG-mediated anticancer effects in PTC. To investigate this, we pretreated PTC cells with NAC, a scavenger of ROS, followed by SNG exposure. BCPAP and TPC-1 cells were pretreated with 10 mM NAC for one hour and then treated with a 4 μM SNG for 4 h. We discovered an integral role of ROS in SNG-mediated cellular alterations and reduced cell viability, as our data suggested that NAC treatment reversed SNG induced morphological changes and inhibition of cell viability in BCPAP and TPC-1 cells, as shown in Figure 4A,C and Figure 4B,D, respectively. In a similar experimental setting, NAC treatment of PCT cells prevented SNG-mediated activation of caspase-3, cleaved caspase-3 and PARP (Figure 4E,F); hence, the data, as presented here, supported the involvement of ROS in SNG induced apoptosis in PTC cells.

### 2.3. SNG Suppresses p-STAT3 and Its Associated Signaling Molecules in PTC Cells

**Figure 5:**
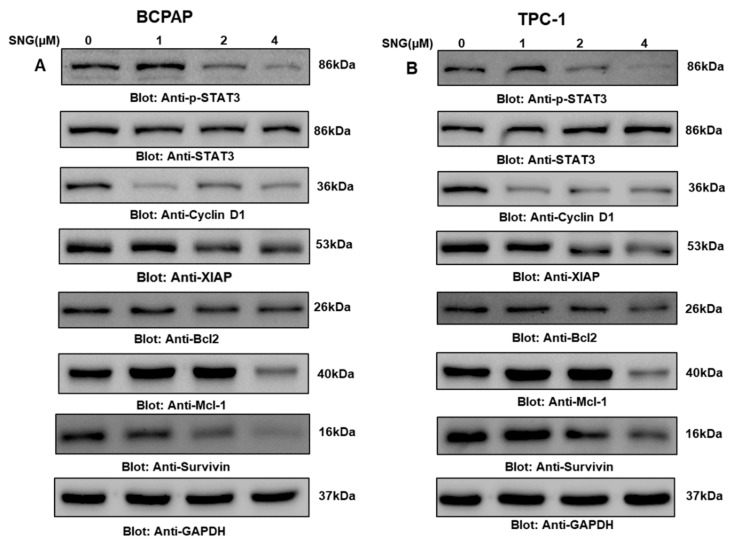
SNG suppresses the constitutively activated STAT3 signaling pathway. BCPAP (**A**) and TPC-1 (**B**) cells were treated with increasing doses of SNG for 4 h, as indicated. After cell lysis, equal amounts of proteins were separated by SDS–PAGE, transferred to a PVDF membrane, and immunoblotted with antibodies of p-STAT3^(Try705)^, STAT3, Cyclin D1, XIAP, Bcl2, MCL1, survivin and GAPDH.

STATs are the transcription factors crucial for biological homeostasis and survival; they function in a coordinated and controlled fashion, and their deregulation results in the development of several pathophysiological conditions. As available clinical and nonclinical data from cancer studies have reported, overexpression of STAT3 has a crucial role in maintaining cancer hallmarks, therefore, in the current study, we explored the status of activated STAT3. For this, we treated PTC cell lines BCPAP and TPC-1 with increasing doses of SNG for 4 h, and the subsequent levels of phosphorylated STAT3 at Tyr-705 were evaluated by Western blotting. As shown in Figure 5A,B, SNG markedly suppressed phosphorylated STAT3 in BCPAP and TPC-1 cells, respectively. Densitometry analysis further supported our data as represented in Supplementary Materials Figure S3A,B (BCPAP and TPC-1, respectively); however, no change was observed in total STAT3 expression in PTC cell lines after SNG exposure, suggesting a change in just the activation of STAT3 in PTC. We also investigated the expression analysis of p-STAT3 and STAT3 at various time points and treated BCPAP cells with 4 μM SNG for 0, 1, 2, 4 and 6 h and found no change in the total STAT3 expression, as shown in Supplementary Materials Figure S2C.

Since STAT3 modulates the expression of genes associated with cell growth and maintenance, we also elucidated the effect of SNG on a number of such genes like Bcl2, XIAP, Mcl-1, survivin and cyclin D1. Western blot data showed that SNG treatment downregulated expression of Bcl2, XIAP, Mcl-1, survivin and cyclin D1, as shown in Figure 5A,B and Supplementary Materials Figure S3D,E. Overall, these findings suggested that modulation of STAT3 signaling played an important role in SNG-mediated inhibition of growth and proliferation of PTC cells.

### 2.4. SNG-Mediated Autophagic Cell Death via ROS Generation

**Figure 6:**
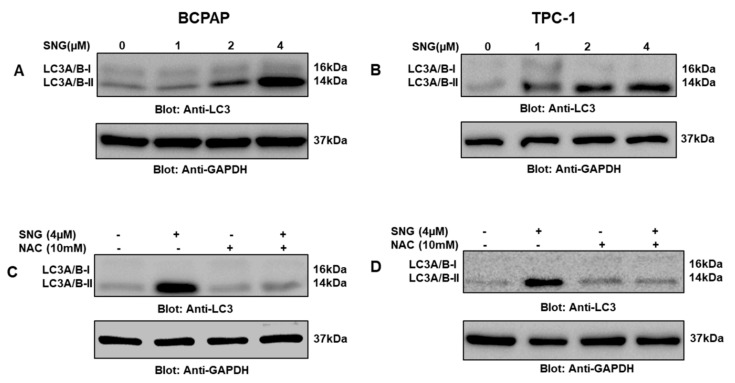
Induction of autophagy in PTC cells treated with SNG. BCPAP (**A**) and TPC-1 (**B**) cells were treated with increasing doses of SNG for 4 h, as indicated. After cell lysis, equal amounts of proteins were separated by SDS–PAGE, transferred to a PVDF membrane, and immunoblotted with antibodies against LC3 and GAPDH. NAC pretreatment of PTC cells prevented SNG-induced LC3 expression. BCPAP (**C**) and TPC-1 (**D**) cells were pretreated with 10 mM NAC and subsequently treated with 4 µM SNG for 4 h. Cells were lysed, and 50 μg of proteins separated on SDS-PAGE, transferred to a PVDF membrane, and immunoblotted with antibodies against LC3 and GAPDH.

Autophagy is another important cellular degradation phenomenon that occurs due to a myriad of stress conditions that play a critical role in cancer biology and associated therapeutic challenges. Therefore, in the present investigation, we wanted to explore whether SNG-mediated inhibition of PTC cell proliferation involved autophagy as well and, if so, its association with ROS. We treated PTC cells with 4 μM SNG with or without 10 mM NAC for 4 h and found that SNG induced upregulation of autophagy marker LC3 while NAC treatment reversed LC3 expression, as shown in Figure 6A–D. This finding suggested that ROS-mediated autophagy played an important role in SNG-mediated death of PTC cells.

### 2.5. SNG Sensitizes PTC Cells to Cisplatin

**Figure 7:**
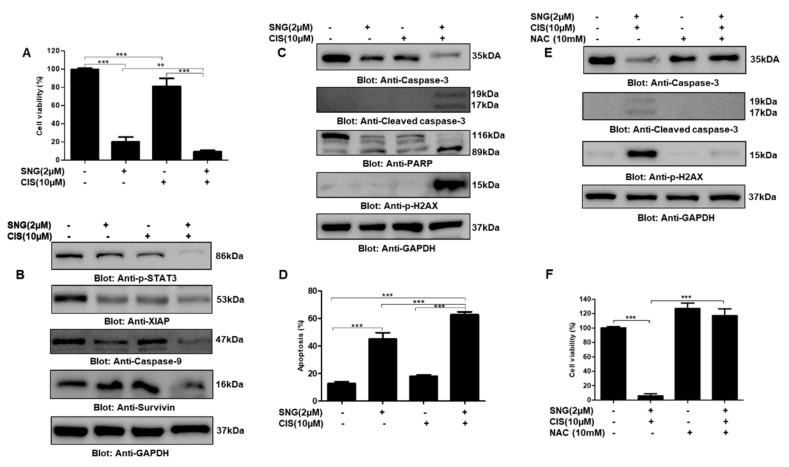
Sanguinarine sensitized PTC cells to anticancer drug cisplatin. (**A**) TPC-1 cells were treated with 2 µM SNG and 10 µM of cisplatin alone and in combination for 24h. CCK-8 was used to determine cell viability, as described in materials and methods. Values are expressed as the mean +/− SD (standard deviation) of at least six replicates with *p* value ** *p* < 0.01, *** *p* < 0.001 (*n* = 6). (**B**,**C**) The combination of SNG and cisplatin inhibits STAT3 phosphorylation and induces apoptosis: TPC-1 cells were treated with 2 µM SNG and 10 µM of cisplatin alone and in combination for 24 h, cells were lysed and separated by SDS-PAGE, transferred to a PVDF membrane, and immunoblotted with antibodies against p-STAT3, XIAP, caspase-9, survivin, caspase-3, cleaved caspase-3, PARP, p-H2AX and GAPDH. (**D**) SNG and cisplatin augmented apoptosis in PTC cells: TPC-1 cells were treated with 2 µM SNG and 10 µM, alone and in combination, for 24 h and processed using annexin V and dead cell kit, according to the manufacturer’s instructions and analyzed by Muse^®^ cell analyzer, as described in materials and methods. The graph displays the mean ± SD (standard deviation) *p* < 0.001 (*n* = 3). ROS plays an important role in SNG-mediated sensitization of PTC cells to cisplatin: (**E**) TPC-1 cells were treated with 10 mM NAC, 2 µM SNG and 10 µM of cisplatin, alone and in combination as indicated, for 24 h. Cells were lysed, and proteins were separated on SDS-PAGE, transferred to a PVDF membrane, and immunoblotted with antibodies against caspase-3, cleaved caspase-3, p-H2AX and GAPDH. (**F**) TPC-1 cells were treated with 10 mM NAC, 2 µM SNG and 10 µM of cisplatin, alone and in combination as indicated, for 24 h. CCK-8 was used to determine cell viability, as described in materials and methods. Values are expressed as the mean +/− SD (standard deviation) of at least six replicates (*n* = 6). *** *p* < 0.001.

There have been many efforts to develop combinational therapies in order to get better clinical outcomes. Keeping this in mind, in this study, we also wanted to know the effect of SNG in combination with chemotherapeutic drug cisplatin on PTC cells. We treated PTC cell line TPC-1 with 2 μM SNG and 10 μM cisplatin alone and in combination for 24 h. CCK-8 based cell viability assay demonstrated that treatment of TPC-1 cells with SNG and cisplatin inhibited cell viability, as compared to alone treatment, as shown in Figure 7A, suggesting that SNG significantly sensitizes PTC cells to cisplatin. To further explore the potential of SNG in the sensitization of PTC cells to cisplatin, we checked the expression of various proteins associated with cell growth and proliferation. Western blot data revealed that a combination of SNG and cisplatin had a profound effect on suppression of p-STAT3, survivin, XIAP and caspase-9, as depicted in Figure 7B. Further, we also observed increased activation of caspases, PARP and DNA double-strand break marker p-H2AX in PTC cells treated with both SNG and cisplatin as compared to alone treatments, as shown in Figure 7C. Annexin V and dead cell kit based cell analysis by Muse^®^ cell analyzer also revealed that combination of SNG and cisplatin induced significantly more apoptosis as compared to individual treatment, as represented in Figure 7D and Supplementary Materials Figure S3F, further supporting the sensitizing potential of SNG to cisplatin. Next, to explore the role of ROS in SNG-mediated sensitization of PTC cells to cisplatin, we treated TPC-1 cells with NAC, SNG and cisplatin, alone and in combination, and performed the expression analysis of apoptotic markers caspase-3, cleaved caspase-3 and p-H2AX. Our data analysis showed that NAC treatment reversed SNG and cisplatin-induced activation of apoptotic markers suggesting that ROS played an important role in the combinational apoptotic effect of SNG and cisplatin in PTC cells, as shown in Figure 7E. We also found that NAC pretreatment significantly prevented SNG and cisplatin-induced inhibition of PTC cell growth and proliferation, as shown in Figure 7F.

### 2.6. SNG Attenuates Stemness Potential of PTC Cells

**Figure 8:**
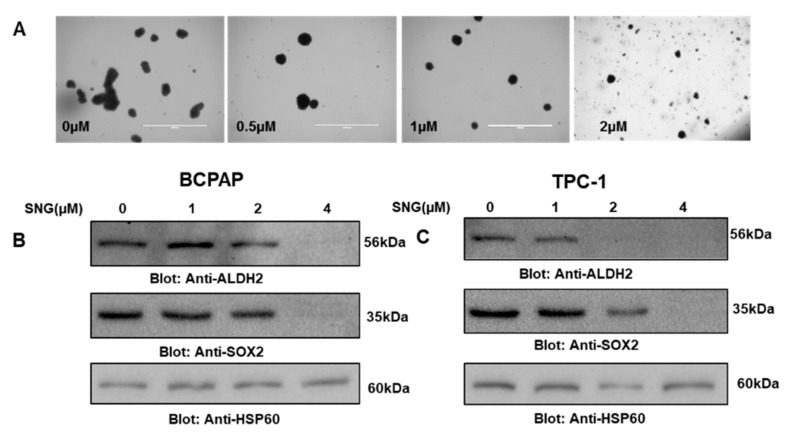
(**A**) SNG-treatment inhibited thyrosphere formation of PTC cells. BCPAP cells were grown and treated in ultralow attachment plates with 0 µM or 0.5 µM, 1 µM and 2 µM of SNG for 7 days, and images of thyrospheres were taken on day 7 using an EVOS FLc cell imaging system from Invitrogen (Thermo Fisher Scientific) at a magnification of 4× (scale bar 1000 µm). (**B**) BCPAP and (**C**) TPC-1 cells spheres were grown and treated in ultralow attachment plates with 0 µM, 1 µM, 2 µM and 4 µM of SNG for 3 days, followed by cell lysis and Western blotting with antibodies against ALDH2, SOX-2 and HSP60.

Cancer stem cells (CSCs) are a small population of cells present in tumors that play a critical role in cancer resistance, relapse, tumorigenesis and poor clinical outcomes [13]. This information led us to investigate whether SNG can suppress the stemness of PTC CSC-like cells derived from BCPAP and TPC-1 spheroids (thyrospheres). An equal number of BCPAP cells were treated with 0, 1.0 and 2.0 µM of SNG for 7 days in ultralow attachment plates, and growth and size of thyrospheres were evaluated. Our data showed that SNG treatment inhibited the size and number of thyrospheres in a dose-dependent manner (Figure 8A). Furthermore, SNG treatment of thyrosphere also suppressed markers of stemness, such as ALDH and SOX-2 in BCPAP (Figure 8B) and TPC-1 (Figure 8C) CSC-like cells. Overall, these findings suggest that SNG treatment of PTC cells not only inhibits the growth and proliferation of cancer cells but also reduces the stemness potential of CSCs.

## 3. Discussion

Natural products have been given special attention to elucidate the underlying mechanisms by which they can act as novel therapeutic drugs. In the present study, we investigated SNG, a natural product usually obtained from the root of *Sanguinaria canadensis*, for its anticancer potential against PTC cell lines, by studying various cellular and molecular alterations underlying PTC pathogenesis. Findings from the current study show that SNG inhibits cell proliferation and growth through ROS mediated apoptosis and autophagy. SNG’s action also involves inhibition of STAT3 activity as SNG treatment suppressed the phosphorylation of STAT3. Furthermore, SNG inhibited cancer stemness potential of PTC cells and sensitized PTC cells to anticancer drug cisplatin.

Uncontrolled cell growth and proliferation are some of the main challenges of cancer pathogenesis, and hence targeting associated deregulated pathways may attenuate oncogenesis. Our results suggest that SNG has potent anticancer potential as it inhibits PTC cell growth through caspase-mediated apoptosis and autophagy. Interestingly earlier reports have shown that SNG has very minimal or no toxic effects on healthy cells [51,52], which is considered a favorable feature for anticancer drug development. Further, our data also revealed that SNG suppressed STAT3 phosphorylation without affecting the total cellular STAT3 protein. STAT3-mediated signaling is often associated with aberrant proliferation and therapeutic resistance of thyroid cancer cells [10,53,54]. Furthermore, STAT3 has been shown to be a major signaling molecule associated with human carcinogenesis, which also causes resistance to cancer therapy and induces stemness [55,56,57,58]; hence, the potential of SNG to suppress the activated STAT3 activity in PTC cells supports its anticancer potential, which is in agreement with previous findings [19,26]. Apoptosis, or programmed cell death, is a vital biological phenomenon essential for homeostasis; it is regulated by a number of signaling proteins, and once deregulated, it leads to the development of various pathological conditions including cancer and therapeutic resistance [59]. Hence, we studied the effect of SNG treatment on the apoptotic markers in PTC cells, and our data reveals that SNG causes activation of caspases and other proteins associated with apoptosis, including downregulation of XIAP and survivin, showing that SNG-mediated inhibition of PTC cell proliferation involves activation of caspases and downregulation of inhibitors of apoptosis proteins. Further, we observed increased p-H2AX expression in PTC cells challenged with SNG, which indicates the role of double DNA strand break, a crucial event for apoptosis execution [60,61]. Furthermore, the use of z-VAD-FMK, z-LEHD-FMK and z-IETD-FMK supports the involvement of caspase-cascades in SNG-mediated apoptosis in PTC cells. Autophagy is another important cellular degradation event associated with cell death, which has been shown to play an important role in cancer pathogenesis and therapy [62,63,64]. We found that SNG induces autophagy in PTC cells, as we detected increased expression of LC3, which is an important autophagic marker; this is in agreement with findings from a previous investigation [65]. Production of ROS due to cancer chemotherapeutics has been shown to play a major role in killing cancer cells [66]. Further, elevated ROS levels in cancer cells usually associate with cancer hallmarks, including genomic instability, and have been considered as the prime target for selective cancer killing [67,68]. Our findings support the critical role of ROS in SNG-mediated PTC cell death as NAC reversed molecular and cellular alterations related to apoptosis and autophagy.

The cotreatment approach has been given attention to achieve better therapeutic outcomes. Our findings revealed that SNG increases the sensitivity of PTC cells to the anticancer drug cisplatin via targeting signaling proteins. Remarkable phosphorylation of H2AX, cleaved PARP, caspase activation and downregulation of survivin and STAT3 in combinational treatment, as compared to alone drugs, provides the molecular basis of SNG-mediated sensitization of PTC cells to cisplatin. In this context, earlier reports also provide additional support for the combinational therapeutic potential of SNG [48,49], indicating that SNG has the potential to affect the growth and proliferation of cancer cells alone and can sensitize malignant cells to cancer therapeutic drugs.

Cancer stem-like cells are the small population of cells within the tumor that are well known for their important role in carcinogenesis, cancer resistance, recurrence and self-renewal [13,69,70]. Among the many laboratory models in use for the study of cancer stem-like cells, the one involving the growth of cancer cells on ultralow attachment plates is a widely accepted model for the generation of spheres. In this current study focused on thyroid cancer, the spheres were named ‘thyrospheres’ to be in sync with the accepted nomenclature in the literature. We report here the SNG-mediated inhibition of thyroid cancer stem-like cells or thyrospheres, which is a good indication of the effectiveness of SNG against the cancer stem-like cells representing a thyroid cancer model. As further proof, our data on downregulation of stemness-related markers, ALDH2 and SOX2, provides additional support for the activity of SNG against cancer stem-like cells.

Although in the present investigation we have demonstrated the in vitro anticancer potential of SNG in PTC, via targeting of a number of molecules associated with cell proliferation and growth and cancer stemness, further studies in appropriate animal models are essentially needed to confirm these results and for the potential future development of SNG as a potent anticancer agent against PTC.

In conclusion, our present study established the anticancer potential of SNG against PTC cells. SNG inhibited PTC cell proliferation through ROS mediated apoptosis and autophagy, and through downregulation of STAT3. SNG also inhibited the thyroid cancer stemness potential and sensitized PTC cells to chemotherapeutic drug cisplatin.

## 4. Materials and Methods

### 4.1. Chemicals and Reagents

Sanguinarine, NAC (N-Acetyl-L-cysteine), and other high-grade reagents were purchased from Sigma–Aldrich (St. Louis, Missouri, USA). Antibodies against caspase3, cleaved caspase3, PARP, p-H2AX, LC3, STAT3, p-STAT3 and GAPDH were procured from Cell Signaling Technologies (3 Trask Lane, Danvers, MA, USA). Antibodies against HSP60, GAPDH, SOX-2 and BCL2 were obtained from Santa Cruz Biotechnology, Inc. (Finnell Street, Dallas, Texas, USA). z-VAD-FMK was obtained from Calbiochem (San Diego, CA, USA). Inhibitors of caspase-8 (Z-IETD-FMK) and caspase-9 (Z-LEHD-FMK) were purchased from R&D system (Minneapolis, Minnesota, USA). Laemmli sample buffer 1X, resolving gel buffer, acrylamide/bis solution, stacking gel solution, developer kit (Clarity Western ECL) were purchased from BIO-RAD (Hercules, California, USA). E-Plate VIEW 16 and CIM-Plate 16 were purchased from ACEA Biosciences (San Diego, California, USA).

### 4.2. Cell Culture

Human papillary thyroid cancer (PTC) cell lines BCPAP and TPC-1 were obtained from DSMZ, Braunschh, Germany, and EMD Millipore, USA, respectively. Cells were cultured in RPMI 1640 medium, supplemented with 10% (*v*/*v*) fetal bovine serum (FBS), 100 U/ml penicillin and 100 U/ml streptomycin, at 37 °C in a humidified chamber containing 5% CO_2_. Treatments were done in 5% FBS-containing RPMI medium.

### 4.3. Cell Counting Kit-8 (CCK-8) Assay

The antiproliferative effect of SNG, on BCPAP and TPC-1 cells, was evaluated by using cell counting kit-8 (CCK-8) assay as described earlier [71]. To do so, we plated 10^4^ cells/well in a 96 well plate and then incubated the plate at 37 °C/5% CO_2_. After 24-hours, cells were treated with five different doses (0.5 µM, 1 µM, 2 µM 4 µM and 8 µM) of SNG for 24 h. The next day, CCK-8 solution was added as per the manufacturer’s instruction, followed by incubation at 37 °C/5% CO_2_. Finally, the optical density (OD) was taken at 450 nm. The percent cell viability was calculated as (OD of the experiment samples/OD of the control samples) × 100.

### 4.4. Real-Time Cell Analyzer (RTCA) Analysis

To observe the growth of PTC cells under different treatment conditions in a real-time, we first plated BCPAP and TPC-1 cells as a monolayer on top of the electrodes, as reported earlier [71], followed by treatment with different doses of SNG. The real-time cell analyzer and E-plate 16 (RTCA; xCELLigence, Roche, San Diego, CA, USA) were used to determine the PTC cell index of SNG treated and untreated cultured cells. To assess the effect of SNG on the migration of PTC cells, first, the lower chamber of the CIM plates was filled with 30% RPMI media and then approximately 30,000 BCPAP cells in 1% RPMI media containing varying doses of SNG were seeded in the upper chamber of the CIM plates. The impedance value of each well was calculated by the xCELLigence system and expressed as cell index.

### 4.5. Cell Cycle Analysis

PTC cells (BCPAP and TPC-1) were seeded in 100 mm culture plates followed by overnight incubation at 37 °C with 5% CO_2_. Next, cells were treated with gradient doses of SNG for 24 h followed by harvesting, centrifugation, fixing in ethanol (70%) and overnight incubation at 4 °C. The next day, cells were resuspended in PI-containing PBS, followed by 30 minutes incubation at 37 °C. Finally, the cells were analyzed by flow cytometry, using a BD LSRFortessa analyzer (BD Biosciences, USA).

### 4.6. Muse^®^ Annexin V and Dead Cell Assay

To quantify the level of apoptosis due to SNG treatment in PTC cell lines, we plated an equal number of cells followed by treatment with varying doses of SNG for about 24 h. Cells were then harvested and washed, and the level of apoptosis was measured on Mouse cell analyzer using Annexin V and dead cell kit (Millipore, USA; cat# MCH100105), as per the manufacturer’s instructions. Further, data collection regarding the number of early and late apoptotic cells was done on Mouse cell analyzer, and total apoptosis was calculated by adding a percent of cells in early and late apoptosis quadrants of the histograms and plotted in bar graphs.

### 4.7. Cell Lysis and Immunoblotting

To check the expression of different proteins, PTC cells were treated with indicated concentrations of SNG, cisplatin and other reagents, followed by cell lysis. Immunoblotting was done, as described earlier [72]. Briefly, proteins were resolved by SDS-PAGE and transferred to a polyvinylidene difluoride (PVDF) membrane, followed by blocking. Next, blots were incubated with specific antibodies raised against the indicated proteins, followed by washing and addition of HRP-conjugated secondary antibody. Finally, blots were visualized under a chemi-doc system, using ECL substrate (Bio-Rad, Hercules, California, USA).

### 4.8. Thyrosphere Cell Culture

PTC cell lines BCPAP and TPC-1 were cultured in complete RPMI media in an incubator with 5% CO_2_ at 37 °C. To investigate the effect of indicated doses of SNG on thyrosphere formation and stemness, BCPAP and TPC-1 cells were cultured and treated with SNG in ultralow attachment plates (Corning, USA) in complete cancer stem cell medium (3D Tumorsphere Medium XF, Promo Cell, Germany, C-28070) in a humidified chamber containing 5% CO_2_ at 37 °C. After the growth for 7 days, spheres were photographed using an EVOS FLc cell imaging system from Invitrogen (Thermo Fisher Scientific) at a magnification of 4× (scale bar 1000 µm).

### 4.9. Statistical Analysis

Results were analyzed using one-way ANOVA followed by a paired Student’s *t*-test using the software GraphPad Prism (version 5.0 for Windows, GraphPad Software Inc., San Diego, CA, USA, http://www.graphpad.com). Values of *p* < 0.05 were considered statistically significant.

## Supplementary Materials

The following are available online. Figure S1: (A) xCELLigence Real-Time Cell Analysis (RTCA) based analysis of cell migration. BCPAP cells were treated with indicated doses of SNG in CIM plates and cell index was monitored as described in materials and methods. (B) BCPAP and (C) TPC-1 cells were cells were treated with indicated concentrations of SNG for 24 h and cells were processed for the cell cycle analysis by flow cytometry as mentioned in materials and methods SNG treatment markedly enhanced SubG0 fraction in PTC cells. (D) BCPAP and (E) TPC-1 cells were cells were treated with indicated concentrations of SNG for 24 h. Then, cells were harvested, washed and level of apoptosis was measured on Mouse cell analyzer using Annexin V and Dead Cell Kit (Millipore, USA cat # MCH 100105) as per the manufacturer’s instructions. Densitometric analysis of p-H2AX in BCPAP (E) and TPC-1 (F) respectively. Cells were treated with increasing doses of SNG for 4 h as indicated in the main text. After cell lysis, equal amounts of proteins were separated by SDS–PAGE, transferred to PVDF membrane and immunoblotted with antibodies against p-H2AX, and GAPDH. Data expressed as relative density normalized with GAPDH and represented as mean ± SD (* *p* < 0.05). Figure S2: (A) Treatment of BCPAP cells with caspase-9 inhibitor Z-LEHD-FMK. BCPAP cells were treated with treated with 30 µM Z-LEHD-FMK and 4 µM SNG alone and in combination for 4 h. CCK-8 was used to determine cell viability as described in materials and methods. Values are expressed as the mean +/− SD (standard deviation) of at least six replicates with *p* value *** *p* < 0.001, ** *p* < 0.01 (*n* = 6). (B) Treatment of BCPAP cells with caspase-9 inhibitor Z-LEHD-FMK. BCPAP cells were treated with 30 µM Z-LEHD-FMK and 4 µM SNG alone and in combination for 4 h followed by assesment of morphological changes by EVOS FLc Cell Imaging System from Invitrogen (Thermo Fisher Scientific). (C) BCPAP cells were treated with 30 µM caspase-9 inhibitor Z-LEHD-FMK and 4 µM SNG alone and in combination for 4 h followed by cell harvesting, lysate preparation, SDS-PAGE separation and immunoblotting with caspase-3, cleaved caspase-3 and GAPDH antibodies. (D) Treatment of BCPAP cells with caspase-8 inhibitor Z-IETD-FMK. BCPAP cells were treated with 30 µM Z-IETD-FMK and 4 µM SNG alone and in combination for 4 h and photomigraphs of cell morphologies were taken. (E) BCPAP cells were treated with 30 µM Z-IETD-FMK and 4 µM SNG alone and in combination for 4 h followed by cell harvesting, lysate preparation, SDS-PAGE separation and immunoblotting with caspase-3, cleaved caspase-3 and GAPDH antibodies. Figure S3: Densitometric analysis of p-STAT3 in BCPAP (A) and TPC-1 (B) respectively. PTC Cells were treated with increasing doses of SNG for 4 h as indicated in the main text. After cell lysis, equal amounts of proteins were separated by SDS–PAGE, transferred to PVDF membrane and immunoblotted with antibodies against p-STAT3, STAT3 and GAPDH. Data expressed as relative density normalized with GAPDH and represented as mean ± SD (* *p* < 0.05). (C). Time course analysis of SNG treatment on PTC cells. BCPAP cells were treated with 4 µM SNG and cells were lysed at different time points as indicated. After cell lysis, equal amounts of proteins were separated by SDS–PAGE, transferred to PVDF membrane and immunoblotted with antibodies against p-STAT3, STAT3 and Actin. Densitometric analysis of cycklin D1 expression (D,E) in BCPAP and TPC-1 cells treated with increasing doses of SNG for 4 h respectively. After cell lysis, equal amounts of proteins were separated by SDS–PAGE, transferred to PVDF membrane and immunoblotted with antibodies against cyclin D1 and GAPDH. Data expressed as relative density normalized with total STAT3, GAPDH and represented as mean ± SD (* *p* < 0.05). (F) TPC-1 cells were cells were treated with indicated concentrations of SNG and cisplatin for 24 h. Then, cells were harvested, washed and level of apoptosis was measured on Mouse cell analyzer using Annexin V and Dead Cell Kit (Millipore, USA cat # MCH 100105) as per the manufacturer’s instructions.
