# Supplementary material for: Sanguinarine Induces Apoptosis in Papillary Thyroid Cancer Cells via Generation of Reactive Oxygen Species
Source: Molecules. 2020 Mar 9;25(5):1229. doi: 10.3390/molecules25051229 (PMC7179475; doi:10.3390/molecules25051229)
Supplement: Supplementary file 1 [file molecules-25-01229-s001.pdf]

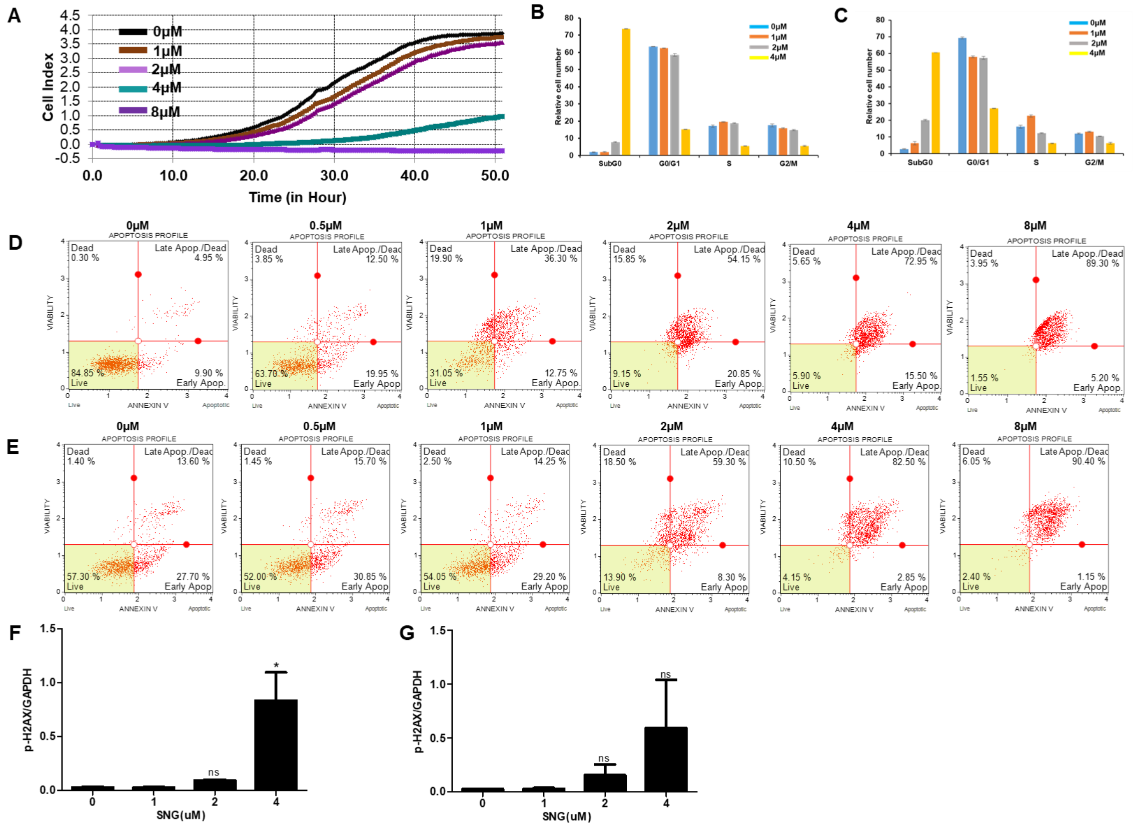

Figure S1: **(A)** xCELLigence Real-Time Cell Analysis (RTCA) based analysis of cell migration. BCPAP cells were treated with indicated doses of SNG in CIM plates and cell index was monitored as described in materials and methods**.** **(B)** BCPAP and **(C)** TPC-1 cells were cells were treated with indicated concentrations of SNG for 24 hours and cells were processed for the cell cycle analysis by flow cytometry as mentioned in materials and methods SNG treatment markedly enhanced SubG0 fraction in PTC cells. **(D)** BCPAP and **(E)** TPC-1 cells were cells were treated with indicated concentrations of SNG for 24 hours. Then, cells were harvested, washed and level of apoptosis was measured on Mouse cell analyzer using Annexin V and Dead Cell Kit (Millipore, USA cat # MCH 100105) as per the manufacturer's instructions. Densitometric analysis of p-H2AX in BCPAP **(F)** and TPC-1 **(G)** respectively. Cells were treated with increasing doses of SNG for 4 hours as indicated in the main text. After cell lysis, equal amounts of proteins were separated by SDS–PAGE, transferred to PVDF membrane and immunoblotted with antibodies against p-H2AX, and GAPDH. Data expressed as relative density normalized with GAPDH and represented as mean ± SD (*p<0.05).

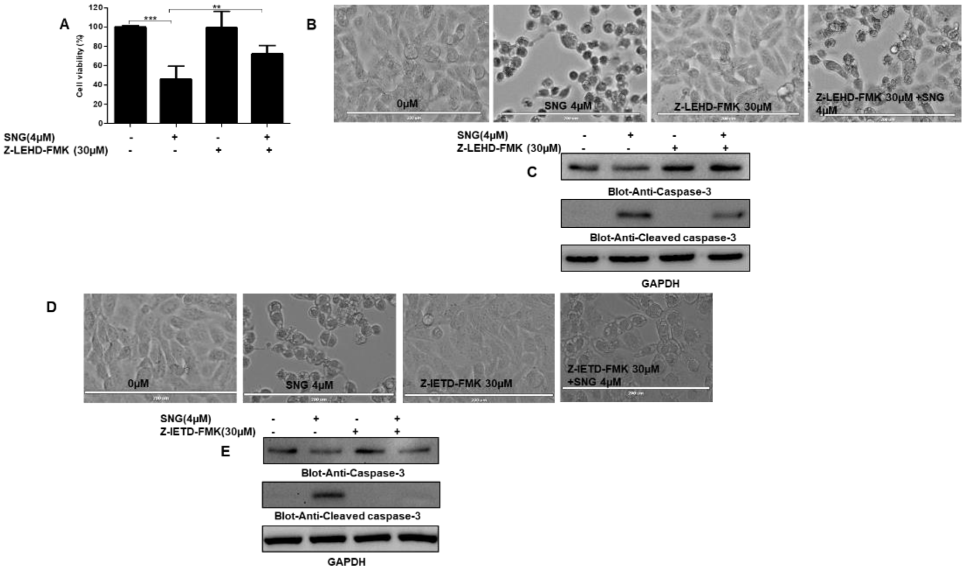

Figure S2: **(A)** Treatment of BCPAP cells with caspase-9 inhibitor Z-LEHD-FMK. BCPAP cells were treated with treated with 30μM Z-LEHD-FMK and 4μM SNG alone and in combination for 4 hours. CCK-8 was used to determine cell viability as described in materials and methods. Values are expressed as the mean +/- SD (standard deviation) of at least six replicates with p value ***p<0.001, **p<0.01 (n=6). **(B)** Treatment of BCPAP cells with caspase-9 inhibitor Z-LEHD-FMK. BCPAP cells were treated with 30μM Z-LEHD-FMK and 4μM SNG alone and in combination for 4 hours followed by assesment of morphological changes by EVOS FLc Cell Imaging System from Invitrogen (Thermo Fisher Scientific). **(C)** BCPAP cells were treated with 30μM caspase-9 inhibitor Z-LEHD-FMK and 4μM SNG alone and in combination for 4 hours followed by cell harvesting, lysate preparation, SDS-PAGE separation and immunoblotting with caspase-3, cleaved caspase-3 and GAPDH antibodies. **(D)** Treatment of BCPAP cells with caspase-8 inhibitor Z-IETD-FMK. BCPAP cells were treated with 30μM Z-IETD-FMK and 4μM SNG alone and in combination for 4 hours and photomigraphs of cell morphologies were taken. **(E)** BCPAP cells were treated with 30μM Z-IETD-FMK and 4μM SNG alone and in combination for 4 hours followed by cell harvesting, lysate preparation, SDS-PAGE separation and immunoblotting with caspase-3, cleaved caspase-3 and GAPDH antibodies.

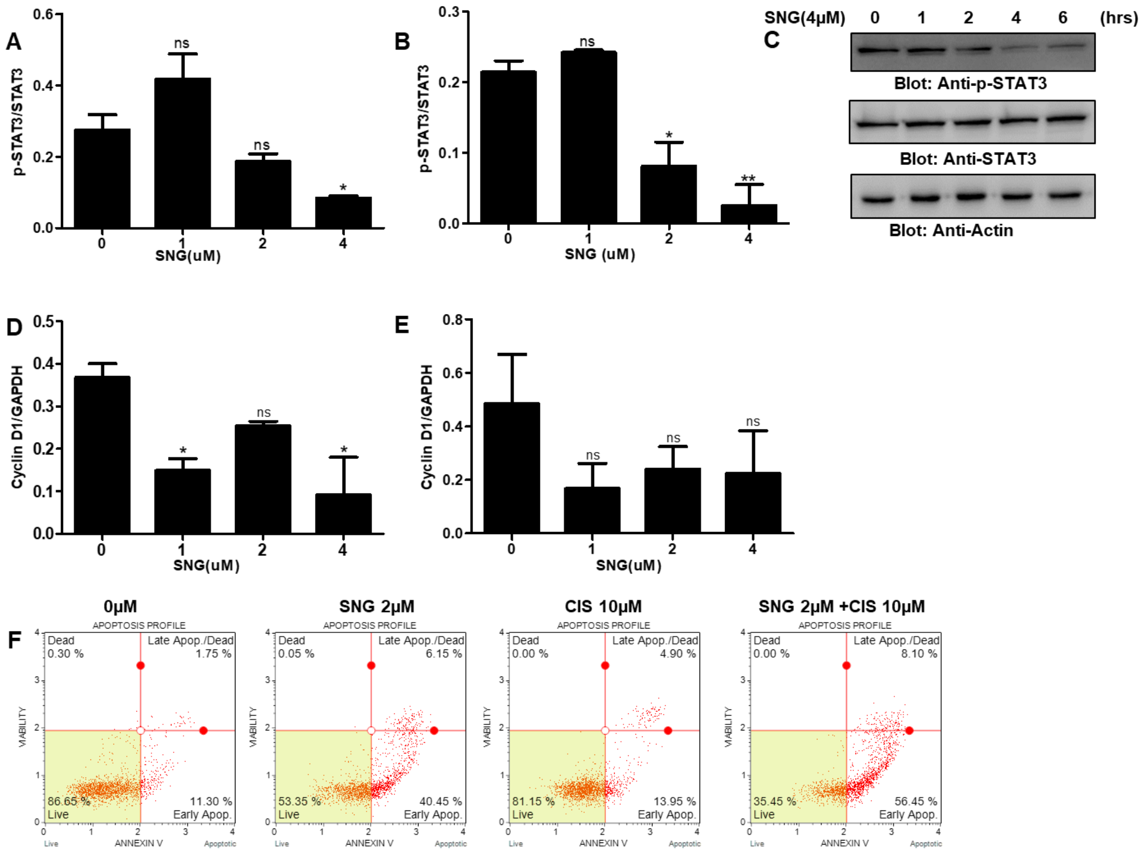

Figure S3: Densitometric analysis of p-STAT3 in BCPAP **(A)** and TPC-1 **(B)** respectively. PTC Cells were treated with increasing doses of SNG for 4 hours as indicated in the main text. After cell lysis, equal amounts of proteins were separated by SDS–PAGE, transferred to PVDF membrane and immunoblotted with antibodies against p-STAT3, STAT3 and GAPDH. Data expressed as relative density normalized with GAPDH and represented as mean ± SD (*p<0.05). **(C)**. Time course analysis of SNG treatment on PTC cells. BCPAP cells were treated with 4μM SNG and cells were lysed at different time points as indicated. After cell lysis, equal amounts of proteins were separated by SDS–PAGE, transferred to PVDF membrane and immunoblotted with antibodies against p-STAT3, STAT3 and Actin. Densitometric analysis of cycklin D1 expression **(D, E)** in BCPAP and TPC-1 cells treated with increasing doses of SNG for 4 hours respectively. After cell lysis, equal amounts of proteins were separated by SDS–PAGE, transferred to PVDF membrane and immunoblotted with antibodies against cyclin D1 and GAPDH. Data expressed as relative density normalized with total STAT3, GAPDH and represented as mean ± SD (*p<0.05). **(F)** TPC-1 cells were cells were treated with indicated concentrations of SNG and cisplatin for 24 hours. Then, cells were harvested, washed and level of apoptosis was measured on Mouse cell analyzer using Annexin V and Dead Cell Kit (Millipore, USA cat # MCH 100105) as per the manufacturer's instructions.
